# Letter to the editor, regarding “Acute care of spontaneous intracerebral haemorrhage”, recently published by Shah and colleagues

**DOI:** 10.1186/s42466-026-00475-7

**Published:** 2026-03-17

**Authors:** Matija Zupan, Mišo Šabovič, Pawel Kermer, Senta Frol

**Affiliations:** 1 Department of Vascular Neurology, University Medical Centre Ljubljana, Ljubljana, Slovenia; 2 Faculty of Medicine, University of Ljubljana, Ljubljana, Slovenia; 3 Department of Vascular Disorders, University Medical Center Ljubljana, Ljubljana, Slovenia; 4 Department of Neurology, University Medical Center Göttingen, Goettingen, Germany; 5 Department of Neurology, Friesland Kliniken GmbH, Sande, Germany; 6 Department of Vascular Neurology, Faculty of Medicine, Ljubljana University, Zaloška 2, Ljubljana, 1000 Slovenia

## Dear editor,

We read with great interest the recent article by Shah and colleagues titled “Acute care of spontaneous intracerebral hemorrhage” presenting a standard operating procedure for the management of acute spontaneous intracerebral hemorrhage (ICH) [1]. The authors are to be commended for delivering a clear, clinically oriented and excellently structured protocol that translates contemporary evidence into practical bedside action. Such initiatives are essential for harmonizing care pathways and reducing unwarranted variability in a condition that remains one of the most devastating neurological emergencies.

For many years, patients with ICH have stood in the shadow of acute ischemic stroke, where the principle of “Time is brain” has long guided systems of care. Growing evidence now indicates that this paradigm is equally relevant for ICH [2]. Recent observational and registry analyses demonstrate that ultrafast diagnostic work-up and immediate initiation of targeted therapies are associated with lower rates of hematoma expansion and improved outcomes [3, 4, 5]. This emerging body of data has led to a conceptual shift advocating “Time is brain” should be fully adopted for ICH as well [2]. The introduction of the “Code ICH” initiative further emphasizes the need for streamlined prehospital notification and in-hospital prioritization [6].

Central to this time-critical approach are acute care bundles that combine rapid blood pressure reduction, prompt reversal of anticoagulation or coagulopathy, early neurosurgical assessment, and standardized neurocritical care. Implementation studies have shown that such bundles improve process metrics and are associated with better functional outcomes [7]. The latest joint European Stroke Organisation and European Academy of Neurology consensus guideline [8] clearly defines treatment goals: immediate lowering of systolic blood pressure toward 130 to 140 mmHg using titratable intravenous agents; urgent reversal of vitamin K antagonists with prothrombin complex concentrates and vitamin K, specific reversal of direct oral anticoagulants when indicated as well as early neurosurgical evaluation including consideration of minimally invasive hematoma evacuation or external ventricular drainage in patients with intraventricular hemorrhage. Parallel advances in mobile stroke units raise the prospect of prehospital ICH diagnosis and initiation of blood pressure control, potentially shortening onset-to-treatment times even further. Avoiding early pessimism can promote favorable outcomes [1]. Accurate prognostic markers are also evolving and may support individualized decision-making [9, 10].

Against this background of therapeutic momentum, the recent withdrawal of the specific factor Xa reversal agent andexanet al.fa (AA) from the United States market creates substantial uncertainty [11]. We believe this issue deserves focused discussion within the stroke community.

AA was developed as a targeted reversal agent for direct factor Xa inhibitors and demonstrated rapid neutralization of anticoagulant activity in clinical trials [12, 13]. However, despite improved hemostatic efficacy in factor Xa inhibitor associated ICH, studies consistently showed increased thromboembolic events without corresponding benefits in mortality or functional outcome [14]. The recent voluntary withdrawal of AA from the US market following an unfavorable benefit to risk reassessment represents a pivotal moment in acute stroke care. This gap between radiographic success and patient centered benefit exposes the limits of surrogate endpoints in acute ICH. Prothrombin complex concentrates (PCCs) remain the principal alternative with modest efficacy and lower reported thromboembolic rates [15].

## Questions for the community

In light of this development, several questions arise. Have current systems of care invested sufficiently in alternatives for factor Xa inhibitor reversal; including optimized PCC protocols and rapid anticoagulant testing? How should guidelines evolve to address the absence of a specific antidote in some regions? What lessons can be learned from regions where AA remains available? How might individualized thrombotic risk assessment inform consideration of AA in carefully selected patients, and what monitoring or safety measures could be implemented to minimize potential risks? Should registries or real-world studies collect more patient-centered outcomes for factor Xa inhibitor–associated ICH and different reversal strategies? Finally, what course might the European Medicines Agency take, balancing residual hemostatic benefits against safety concerns of AA, and could conditional restriction to highly selected scenarios be a possible path? In our view, future priorities should include optimized PCC strategies, novel antidotes, thrombotic risk stratification, and rapid point of care testing. Factor XI or XIa inhibitors may offer safer anticoagulation and reduce the need for reversal [16]. The US withdrawal of AA underscores that reversal must deliver meaningful outcomes and renewed patient centered research is essential.

We congratulate the authors once again for their timely SOP and believe that addressing these unresolved issues will be crucial to fully realizing the “Time is brain” concept for patients with ICH.

## Data Availability

The dataset is available on request from the authors.
